# Structural and functional alterations in photosynthetic apparatus of plants under cadmium stress

**DOI:** 10.1186/1999-3110-54-45

**Published:** 2013-10-08

**Authors:** Pooja Parmar, Nilima Kumari, Vinay Sharma

**Affiliations:** Department of Bioscience and Biotechnology, Banasthali University, P.O. Banasthali Vidyapith, Rajasthan, 304022 India

**Keywords:** Cadmium, Photosynthesis, Chlorophyll, Chloroplast, PSII, PSI

## Abstract

Cadmium is a potentially toxic heavy metal that hampers plant productivity by interfering with their photochemistry. Cd causes disturbances in a range of physiological processes of plants such as photosynthesis, water relations, ion metabolism and mineral uptake. Cd pronouncedly affects photosynthesis by alteration of its vital machinery in all aspects. Photosynthesis is a well organised and sequential process fundamental to all green plants and microorganisms which involves various components, including photosynthetic pigments and photosystems, the electron transport system and CO_2_ reduction pathways. Any damage at any level caused by Cd, critically affects overall photosynthetic capacity. Present review focuses on key effects of Cd on photosynthetic apparatus including chloroplast structure, photosynthetic pigments, Chl-protein complexes and photosystems resulting in overall decrease in efficiency of carbon assimilation pathway.

**Electronic supplementary material:**

The online version of this article (doi:10.1186/1999-3110-54-45) contains supplementary material, which is available to authorized users.

## Review

### Introduction

The unprecedented increase in heavy metal pollution has become a matter of major concern over the globe (Jamali et al. 2007). Cadmium (Cd) stands 7th out of the 20 toxins and has no known biological function except in marine diatoms (Morel 2008). Cd is used and traded internationally as a metal and as a chemical compound throughout Asia, America, Europe, Australia and Africa (UNEP 2010). Indeed, Cd concentration is progressively increasing at an alarming rate (7 to 43 percent over the period of 100 years) in several European countries such as Austria, Denmark, Finland, Greece, Ireland and the United Kingdom (UNEP 2010). It has been estimated that major source of Cd release into the air are the production of nonferrous metals followed by iron and steel production, fossil fuel combustion, cement production and waste incineration (Pacyna and Pacyna 2001). Cd is constantly added and gets accumulated to the plough layer of soil through various natural and anthropogenic activities such as volcanic eruptions, mining, smelting, mismanagement of industrial waste and use of phosphate fertilizers (Grant 2011) and its addition to the arable land is a widely recognised problem. Cd is potentially toxic to all organisms including plants, animals and humans as well. Cd exposure, for instance, is associated with cancers of the prostate, lungs and testes, kidney tubule damages, rhinitis, emphysema, osteomalacia and bone fractures in humans (Nawrot et al. 2006). In plants, it results in many toxic symptoms such as inhibition of growth and photosynthesis, activation or inhibition of enzymes, disturbances in plant-water relations and ion metabolism, and formation of free radicals (Valentoviova et al. 2010).

**Figure 1:**
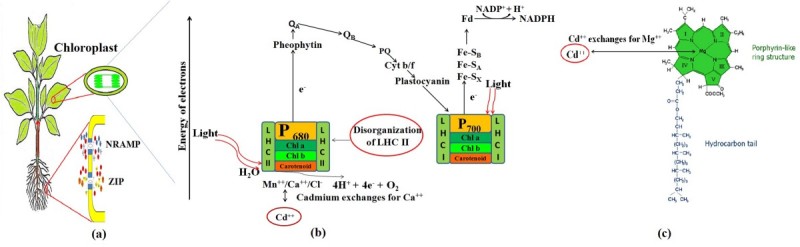
**An overview of effects of Cd exposure to plants at different levels in photosynthetic machinery. (a)** Cd uptake in cells through plasma membrane transporters. **(b)** Alteration in organisation of oxygen evolving and light harvesting complexes, Cd also binds with Q_B_ pocket thus slows down electron flow from Q_A_ to Q_B._**(c)** Incorporation of Cd in chlorophyll molecule.

Phytotoxicity induced by Cd has been well established and comprehensively studied (Wahid et al. 2009). Cd is taken up by roots through plasma membrane transporters such as ZIP (ZRT-IRT like protein; Zinc regulated transporter, Iron-regulated transporter) and NRAMP (natural resistance associated macrophage protein) in competition to the essential nutrients of plants (Kim et al. 2002) and consequently it is translocated to shoots thereby leading to growth diminution which in due part emanates from disturbed photosynthesis (Bazzaz et al. 1974). Figure 1 illustrates the effects of Cd as a potent inhibitor of photosynthesis. Photosynthesis inhibition may be attributed to diminished chlorophyll biosynthesis (Shukla et al. 2008), interrupted O_2_ - evolving reactions of PSII and altered electron flow around PSI and PSII (Mallick and Mohn 2003). Cd hampers Calvin cycle by slowing down activity of various enzymes hence resulting in decreased photosynthesis (Ying et al. 2010). Cd has also been known to show inhibitory effect on various enzymes such as ribulose-1,5-biphosphate carboxylase oxygenase (Mobin and Khan 2007), phosphoenolpyruvate carboxylase (Latif 2008), aldolase (Sheoran et al. 1990), fructose-6-phosphate kinase (Malik et al. 1992), fructose-1,6-bisphosphatase (Sheoran et al. 1990), NADP^+^-glyceraldehyde-3-phosphate dehydrogenase (Sheoran et al. 1990) and carbonic anhydrase (Mobin and Khan 2007).

Stomatal closure due to entry of Cd into the guard cells in competition to Ca^+2^ (Perfus-Barbeoch et al. 2002) and reduction in stomata count per unit area are also characteristic symptoms of Cd stress resulting in lesser conductance to CO_2_ (Pietrini et al. 2010) which consequently leads to overall inhibition of photosynthesis.

The present review is an attempt to develop an orchestrated understanding of the mechanisms involved in altering and damaging various components of photosynthetic machinery by Cd thereby leading to effective loss in the anabolic reactions of plants.

### Photosynthetic machinery under Cd stress

#### Chloroplast structure

Cd convincingly resulted in marked distortion of chloroplast ultrastructure leading to disturbed shape and inflated thylakoids (Najeeb et al. 2011). Chloroplast structure disturbance has been partly manifested by a notable decrease in chloroplast number and size, grana stacking, starch grain content and accumulation of plastoglobuli observed in various plants such as *Picris divarticata* (75 μM, 14 days after treatment (DAT)), *Hordeum vulgare* (5 μM, 15 DAT) and *Brassica* (Ying et al. 2010; Wang et al. 2011; Elhiti et al. 2012). Further, plants show differential aggregation of grana in young and older leaves. For instance in willow, older leaves showed swollen but organised thylakoids whereas young leaves appear to be more dense structured accompanied by tannin precipitation. Reed plant chloroplasts displayed a disturbed shape, wavy appearance of grana and stroma thylakoids and swollen intra thylakoidal space owing to lipid peroxidation, a consequence of increased lipid accumulation in thylakoids (Hakmaouia et al. 2007).

Disruption in chloroplast structure is also ensued due to increased peroxidation of membrane fatty acid and lipid contents resulting from enhanced lipooxygenase (LOX) activity (Remans 2010). LOX mediates polyunsaturated fatty acid oxidation including chloroplast membrane lipids such as monogalactosyldiacyl-glycerol (MGDG), digalactosyldiacyl-glycerol (DGDG) and phosphatidyl glycerol (PG) hence resulting in production of free radicals. LOX activity has been positively correlated with increased lipid peroxidation in plants such as *Arabidopsis*, Barley, Lupine and *Phaseolus* under Cd stress (Maksymiec and Krupa 2006; Tamas et al. 2009). A significant decrease has also been reported in the content of polaracyl lipids especially MGDG, DGDG and PG in tomato chloroplasts membranes (Djebali et al. 2005) which is considered to be indispensable for maintenance of membrane integrity.

Grana disorganization can be attributed to reduced MGDG level, as well as the decrease in 16:1 trans fatty acid content in MGDG and PG. In *Brassica napus* (50 μM, 15 DAT) leaves, remarkable decrease upto 80–84 % was observed in DGDG and MGDG respectively (Nouairi et al. 2005), which may possibly be a reason in disintegrated grana.

#### Cd induced pigment changes

Among the photosynthetic pigments enormous studies have been conducted till date on reduction in chlorophyll and carotenoids in plants exposed to Cd stress. Chlorophyll destruction in older leaves and its biosynthesis inhibition in newer ones have been known to be prime cause in leaf chlorosis in plants growing in Cd treated soils (Xue et al. 2013). Inhibition of chlorophyll biosynthesis enzymes and activation of its enzymatic degradation plays crucial role in net loss in chlorophyll content (Somashekaraiah 1992).

Aminolevulinate (ALA) is a crucial compound in chlorophyll biosynthesis and its synthesis is the rate-limiting and regulatory step. Cd inhibits ALA synthesis at the site of availability of glutamate for ALA synthesis and interferes by interacting with SH group of enzymes, δ-aminolevulinic acid dehydratase (Mysliwa-Kurdziel and Strzalka 2002) and porphobilinogen deaminase, (Skrebsky et al. 2008) leading to the accumulation of chlorophyll biosynthesis intermediates like ALA and porphyrins. In fact ALA accumulation is considered to be a reason for generation of reactive oxygen species which alters redox status of plants and thus disturbing plant homeostasis as reported in *Soybean* (0-100 μM, 10 DAT) and *Cucumis* (0-1000 μM, 10 DAT) (Noriega et al. 2007; Goncalves et al. 2009). Additionally Cd reacts with protochlorophillide reductase, which causes photoreduction of protochlorophillide into chlorophyllide thus diminishing the raw material for chlorophyll synthesis (Stobart et al. 1985).

Cd also decreases uptake of nutrients such as Mn, Fe and Mg, hence a comparatively higher amount of cellular Cd interferes with Mg^2+^ insertion into protoporphyrinogen or may cause Chl destruction as consequence of Mg^2+^ substitution in both Chl a and b (Gillet et al. 2006).

Carotenoid content in plants exposed to Cd do not exhibit a set pattern and may either increase or decrease. The increase has been observed in many cases as in *Cucumis sativus* L. (Burzynski et al. 2007) and *Zea mays* L. (100 μM, 10 DAT) (Chaneva et al. 2010). On the contrary decrease was also observed in a few cases e.g. *Pisum sativum* (7 mg/kg, 20 DAT) (Hattab et al. 2009). Other leaf pigments including neoxanthin, lutein, violaxanthin were found to decrease in *Lycopersicon esculentum* and *Spinacea oleracea* plants (López-Millán et al. 2009; Fagioni et al. 2009).

In lower organisms, Cd exposure caused a significant drop in the amounts of phycobiliprotein viz. allophycocyanin, phycocyanin, and phycoerythrin e.g. *Chlamydomonas* (50 μM, for 24 hrs), *Gracilaria* (300 μM, 16 DAT) and *Hypnea musciformis* (300μM, 7DAT) that led to decrease in photosynthetic efficiency (Perrault et al. 2011; Santos et al. 2012; Bouzon et al. 2012).

#### Cd induced changes in chlorophyll-proteins complexes

Chl-proteins can be described as Chl a and Chl a/b multicofactor proteins for both photosystems (PS) bound to chlorophylls and carotenoids (Fromme et al. 2001). Cd effects on both the PS as well as degree of damage vary in the plant species even among cultivars and populations, depending on genotypic and ecotypic differences (Prasad 1995).

##### PSII core complex

Immunoblotting of Chl-protein complexes did not depict any changes in the level of polypeptides of PSII complexes comprising of CP 47, CP 43, D1 and D2 under Cd stress as demonstrated in rice (75 μM Cd, 28 DAT) and spinach (100 μM Cd, 30 DAT) (Pagliano et al. 2006; Fagioni et al. 2009). The same pattern was also observed in lower organisms i.e. *Chlamydomonas reinhardtii* (50 μM, for 24 hrs) too (Perreault et al. 2011).

Cd toxicity may be attributed to both acceptor and donor side of PSII thus preventing photoactivation (Sigfridsson et al. 2004). On the donor side due to high affinity, Cd exchanges with Ca^++^ in Mn^++^/Ca^++^ cofactor present in oxygen evolving complex (Faller et al. 2005; Pagliano et al. 2006); the exchange leads to reduced kinetics of Hill reaction. On acceptor side Cd decreased the rate of electron transfer from Q_A_ to Q_B_ due to interaction with nonheme Fe and conformational modification of Q_B_ pocket (Geiken et al. 1998).

Further decrease in lipid content in chloroplasts specifically MGDG and DGDG (Nouairi et al. 2005), considered to be indispensable for PSII activity, causes structural disorganisation of PSII supramolecular structure (Quartacci et al. 2000).

##### Light harvesting complex (LHC) II

LHCII is the principle light harvesting pigment-protein complex of PSII which absorbs light energy and transfers it to the reaction centre. The native form of LHCII is a trimer composed of three Lhcb proteins: Lhcb1, Lhcb2 and Lhcb3 (Lucinski and Jackowski 2006). These LHCII aggregates play dynamic role in triggering the thermal dissipation of extra energy for efficient excitation quenching and display photoprotective role in case of overexcitation of reaction centre and antenna (Barros et al. 2009). Cd exposure results in dissipation of total mass of Lhcb1 and Lhcb2 and accounts for disorganization of trimer-forming monomers resulting in diminished LHCII aggregation complexes. This was indicated by infrared studies on *Secale cereale* exposed to Cd (50 μM, 7 DAT) where aggregate/trimeric ratio remained 73% of the control (Janik et al. 2010). Cd toxicity resulted in constrained dissipation of excitation energy which may have been induced by alterations in the quenching centre formation or inhibition of vibrational transfer of thermal energy between pigments and the protein skeleton (Gruszecki et al. 2009). In *Spinacia oleracea* L. Lhcb1.1 isomers of Lhcb1 were highly affected even in small exposure to stress (75 μM, 5 DAT) whereas others i.e. Lhcb2 and Lhcb3 were less affected (Fagioni et al. 2009). Differential level of expression in Lhcb2 was observed in case of two ecotypes of *Sedum* (hyperaccumulating and non-hyperaccumulating) which suggested temporal regulation of gene expression. Upon 24 hrs of Cd (2μ M) treatment non-hyperaccumulating ecotype exhibited higher expression level than hyperaccumulating, followed by a reversal of the situation after 8 days (Zhang et al. 2011).

Proteomic studies on *Oryza sativa* L. (7.5-75μM, 24 DAT) suggested contrasting results where LHCII content is not adversely affected suggesting that antenna complexes of PSII are less affected (Pagliano et al. 2006).

Lipid profiling of chloroplast is conducive to suggest that decrease in 16:1 trans fatty acid content in MGDG and PG, diminished LHCII oligomerization due to its specific binding in sn-2 position in the chloroplastic PG (Vassilev et al. 2004). Cadmium due to its high affinity gets substituted in pigment protein complexes causing conformational changes (Küpper et al. 2002) leading to incorrect binding of chlorophyll molecule to the protein matrix.

##### PSI core complexes

In some plants exposed to cadmium stress PSI instead of PSII is the prime site of damage. Previous studies suggested that Cd induced iron deficiency in cell organelles is possibly a reason for greater damage to PSI (Siedlecka and Baszyński 1993; Timperio et al. 2007). Prolonged deficiency of iron resulted in generation of reactive oxygen species in thylakoids which principally destroys iron-sulphur centres (PSI) and Lhca antennae (Michel and Pistorius 2004). In fact, observations suggesting damage to PSI have been reported in *Cucumis sativus* L. (10 μM, 35 DAT) (Sárvári 2005; Sárvári et al. 2008) and wheat (Atal et al. 1991). However in *Pisum sativum* extended stress treatment of Cd (0–10 mM, 12 DAT) led to equal damage to both PSI and PSII (Chugh and Sawhney 1999).

Proteomic and expression studies conducted on basal leaves of *Spinacia oleracea* L. (100 μM, 0–15 DAT) revealed presence of modified amino acids in polypeptide chains of PsaA/PsaB proteins corroborating the accumulation of incomplete monomeric units leading to disruption of PSI supercomplexes, reaction centre I and LHCI (Fagioni et al. 2009).

#### Cadmium induced changes in photosynthetic yield

The main effect of Cd, studied to date is hampering the photochemical activity of both PSI and PSII. The reports regarding this have always been contradictory in deciding the principal site of damage i.e. PSI or PSII. As observed in peas, Cd affects both the photosystems over a long period of stress (Chugh and Sawhney 1999). However, during initial stages Cd had more pronounced effect on the activity of PSII as observed in *Thalspi caerulescence* (Kupper et al. 2007) stating higher sensitivity of PSII to Cd toxicity (Wang et al. 2013).

The chlorophyll fluorescence induction parameters represent the use of non-invasive tool to understand the photosynthetic performance in vivo and to assess effects of stress on plants photochemistry (Baker et al. 2008). Table 1 depicts effects of Cd on some of the photosynthetic parameters [Chl (chlorophyll content), Fv/Fm (maximum quantum efficiency of PSII) and P_N_ (Net photosynthetic rate)] which reflect lesions in plants photosynthetic yield as a consequence of damage to the photosystems and pigments (Wang et al. 2009). It is evident from the Table 1 that Cd decreased chlorophyll content, F_V_/F_M_ and P_N_ indicating impeded photosynthesis. However, PSII disruption as a consequence of Cd toxicity is reported to depend on the irradiance conditions. During high light intensity, direct damage to the PSII reaction centre occurs instead; this was termed as the 'sun reaction’. On the contrary, LHCII disruption due to exchange of Mg^2+^ with Cd in chlorophyll pigment is the prime cause of diminished PSII activity during dark phase and is referred as 'shade reaction’ (Kupper et al. 2007).

**Table 1:** **Effects of Cd on photosynthetic parameters** ^**1**^

Plant species	Cd concentration	% inhibition				Reference
		Chl a	Chl b	Fv/Fm	Pn	
*Pisum sativum*	6 mM, 7 DAT	31.00	32.30	--*	79.90	; Januškaitienė, 2012
*Picris divarticata*	75 μM, 10 DAT	10.84	1.29	--*	44.20	Ying et al. 2010
*Zea mays*	20 μM, 7 DAT	36.71	37.93	3.76	13.00	Wang et al. 2009
*Ricinus communis*	50 μM, 12 DAT	27.30	16.70	5.00	47.00	Liu et al. 2011
*Lycopersicon esculentum*	100 μM, 12 DAT	35.00	23.00	2.47	73.00	;López-Millán et al. 2009
*Cucumis sativus*	50 μM, 48 hrs	13.89	24.44	1.57	73.00	; Burzynski & Zurek, 2007
*Phragmites australis*	100 μM, 21 DAT	52.30	73.00	1.28	40.88	Pietrini et al. 2003

* Values are not available.

^1^Effective concentration of Cd causing reduction in chlorophyll pigments viz. Chl a and Chl b and non-invasive parameters viz. photosynthetic yield (Fv/Fm) and net CO_2_ assimilation rate (Pn).

Comparatively less information is known to us in case of PSI but still data exists which shows higher sensitivity to PSI photochemistry as in *Pisum sativum* plants (Wodala et al. 2012).

## Conclusion

In conclusion, Cd affects photosynthesis either directly or indirectly thus decreasing the crop yield. We reviewed its inhibitory effect on pigments, lipids, photosystems proteins and chloroplasts. Summing up all we investigated net loss in photosynthesis. It can be said that much has been known about Cd toxicity to plants but numerous mechanisms remains debatable about its interaction with photosynthetic proteins i.e. D1 and D2 and oxygen evolving complexes. In particular, we should extend our knowledge towards PSI measurements to get an intricate knowledge on effect of Cd on photosynthesis. Strategies must be evolved on understanding the mechanism of Cd hyperaccumulation to uphold various phytoremediation strategies.

## Authors’ original submitted files for images

Below are the links to the authors’ original submitted files for images. Authors’ original file for figure 1

## Abbreviations

Cd: Cadmium
ZIP: ZRT-IRT like protein
ZRT: Zinc regulated transporter
IRT: Iron-regulated transporter
NRAMP: Natural resistance associated macrophage protein
MGDG: Monogalactosyldiacyl-glycerol
LOX: Lipooxygenase
DGDG: Digalactosyldiacyl-glycerol
PG: Phosphatidyl glycerol
DAT: Days after treatment
ALA: Aminolevulinate
PS: Photosystems
LHC: Light harvesting complex.
