# Supplementary figures and images for: Structural and functional alterations in photosynthetic apparatus of plants under cadmium stress
Source: Bot Stud. 2013 Oct 8;54:45. doi: 10.1186/1999-3110-54-45 (PMC5430381; doi:10.1186/1999-3110-54-45)

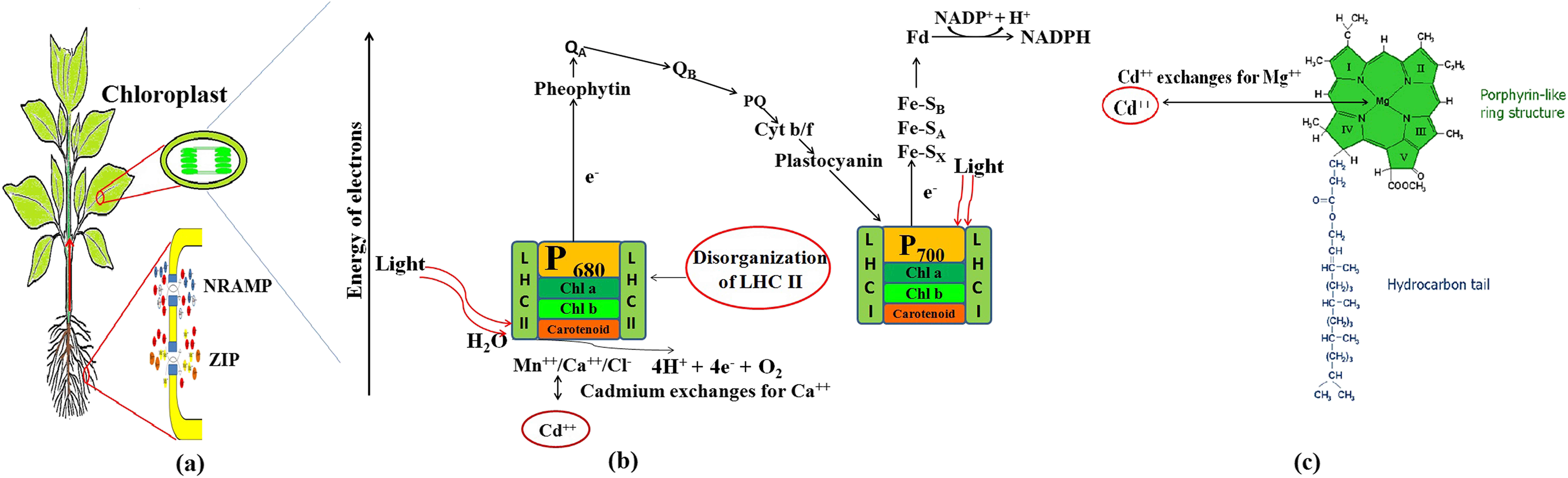

Supplement: Supplementary file 1 — Authors’ original file for figure 1 [file 40529_2013_39_MOESM1_ESM.tif]
